# Neuromuscular activation following anti-movement and dynamic core training: a randomized controlled comparative study

**DOI:** 10.1007/s00421-025-05768-4

**Published:** 2025-04-07

**Authors:** Fahri Safa Cinarli, Muhammed Emin Kafkas

**Affiliations:** Faculty of Sport Science, Inonu University, Malatya, Turkey

**Keywords:** Resistance training, Core training, Electromyography, Muscle activation, Spine

## Abstract

**Background:**

This study investigated the effects of anti-movement and dynamic core training on neuromuscular activation in trained men using surface electromyography (sEMG).

**Methods:**

Thirty-six participants were randomized into the anti-movement (AMG), traditional dynamic (TDG), or control (CG) groups in a controlled study. Training groups performed core exercises twice weekly for 6 weeks, with standardized time under tension (AMG: 30-s isometric holds; TDG: 30 s with 12 repetitions). Anterior and posterior trunk muscle activation was measured using sEMG during isometric endurance tests pre- and post-intervention.

**Results:**

Significant time and interaction effects were observed for lumbar erector spinae (LES) activation (*F* = 3.784, *p* = 0.029), with AMG (*p* = 0.016) and TDG (*p* = 0.004) outperforming CG. A significant enhancement in external oblique (EO; *η*^2^ = 0.17, *p* = 0.023) and internal oblique (IO; *η*^2^ = 0.30, *p* = 0.003) activation was observed in the AMG compared to the CG. Both training groups improved LES (*η*^2^ = 0.37, *p* = 0.001) and multifidus (*η*^2^ = 0.19, *p* = 0.016) activation (*p* < 0.017). Within-group analysis showed significant pre-to-post improvements across all muscles (*p* < 0.05, effect size *r* = 0.48–0.63). Greater reductions in muscle activation (22.17%–53%) were demonstrated by the AMG compared to the TDG (16.18%–28.82%), suggesting improved neuromuscular efficiency.

**Conclusion:**

Anti-movement core training effectively enhances trunk muscle neuromuscular activation and efficiency, providing a robust alternative to traditional protocols.

## Introduction

Core exercises have been a cornerstone of rehabilitation and athletic performance enhancement for more than 25 years, frequently prescribed by physicians and trainers to improve functional outcomes and optimize physical capabilities (Clark et al. 2018). While the development of athletic performance remains a primary objective of core stabilization and strength training programs, growing attention has been directed toward ensuring spinal protection and promoting safe movement patterns during training (McGill 2004).

Traditional core training, involving movements such as flexion, extension, and rotation, has long been recognized for its effectiveness in enhancing strength and stabilization (Schilling et al. 2013). However, concerns have been raised about its potential risks, particularly in relation to spinal health. Repeated flexion and bending movements have been associated with increased pressure on intervertebral disks, potentially leading to conditions such as disk protrusions or herniations (Tampier et al. 2007; Desmoulin et al. 2020). High-repetition loading of the lumbar spine during such movements has also been linked to an elevated risk of injury (Solomonow 2012; McGill 2010a).

Deviations beyond the spine’s safe range during bending, twisting, and rotational movements may compromise the neutral zone, increasing the likelihood of unsafe movement patterns (Yue et al. 2007). The neutral zone represents the optimal physiologic range of controlled motion for the lumbar spine, within which minimal resistance and stress on passive structures are observed (Panjabi 1992). In response to these risks, anti-movement exercises have emerged as a promising alternative, emphasizing the importance of resisting external forces that could displace the spine in any direction (sagittal, frontal, or transverse axes). McGill (2010b, a) defines anti-movement exercises as the capacity to resist motion by generating sufficient moment forces to stabilize the core region, rather than facilitating movement itself. From an etiological perspective, anti-movement exercises were developed to address the need for movement patterns that prioritize spinal protection and reduce the risk of disk-related injuries (Callaghan and McGill 2001). These exercises operate on the principle of resisting movement while simultaneously maintaining stability, aligning with a biomechanical approach that preserves the spine’s neutral zone.

Surface electromyography (sEMG) is a valuable tool for evaluating muscle activation patterns and the recruitment of motor units during exercise. Studies have suggested that reductions in EMG amplitude post-training may reflect improved neuromuscular efficiency and energy utilization (Oliveira and Gonçalves 2009). Greater difficulty in a movement task typically increases motor unit recruitment and EMG activation (Mathur et al. 2005; Cao et al. 2017). However, when training adaptations lead to lower maximal voluntary contraction (MVC) values, it may indicate enhanced training effectiveness and reduced energy expenditure for the same task.

Despite the growing interest in anti-movement training, there remains a significant gap in the literature regarding its specific neuromuscular effects. Previous studies have largely focused on traditional core-training methods, leaving the potential advantages of anti-movement exercises for trunk muscle activation and spinal stability underexplored. Most notably, no research to date has directly compared anti-movement and dynamic core training using standardized electromyographic assessments, creating a clear need for empirical evidence to support the growing clinical and athletic application of anti-movement protocols.

To our knowledge, this is the first study to analyze the activation patterns of anti-movement exercises. We hypothesized that anti-movement training would lead to significantly greater improvements in trunk neuromuscular activation compared to traditional dynamic core training, which incorporates flexion, extension, and rotation movements. Such findings would provide valuable insights for fitness professionals and therapists, highlighting the potential of neutral zone-based training to surpass traditional core training in enhancing trunk muscle performance and spinal health. The aim of this study was to compare the effects of two distinct core-training modalities (anti-movement vs. traditional dynamic) on neuromuscular activation in recreationally trained men.

## Materials and methods

### Experimental design and participants

This study employed a randomized controlled design to evaluate the effects of two core-training modalities on neuromuscular activation. Participants were recreationally trained men who were randomly assigned to one of three groups: the anti-movement group (AMG), the traditional dynamic group (TDG), or the control group (CG). The randomization process was conducted using the block randomization method to ensure balanced allocation across the groups.

Participants were included in the study if they were male, aged between 18 and 35 years, engaged in recreational-level physical activity (2–4 sessions per week), but had not participated in any systematic core strength training. In addition, individuals with no history of chronic lower back pain, neuromuscular disorders, or musculoskeletal injuries within the last six months were eligible to participate. Participants were excluded if they had a history of core-specific strength or power training within the previous six months, any cardiovascular, metabolic, or neurologic conditions, or if they were using performance-enhancing supplements or medications that could affect neuromuscular function. The study adhered to ethical principles outlined in the Declaration of Helsinki and received approval from the university’s Clinical Research Ethics Committee (protocol number: 2019/101). Written informed consent was obtained from all participants prior to their involvement in the study.

### Power analysis

To the best of our knowledge, no previous studies have examined neuromuscular activation patterns following anti-movement exercises. Therefore, we estimated sample size based on Saeterbakken et al. (2018), who investigated dynamic and isometric training effects on core strength. A power analysis (G*Power 3.1.9.3) using α = 0.05, power (1-β) = 0.80, and an effect size of 0.50 indicated that at least 27 participants were required. To account for potential dropouts, we included 36 participants.

### Body composition measurements

All measurements were conducted with participants wearing minimal clothing and barefoot. Height was measured to the nearest 0.1 cm using a portable stadiometer (Seca Ltd., Bonn, Germany) with participants standing upright, in the Frankfort plane, and weight evenly distributed on both feet. Body weight and body fat percentage were assessed using a body composition analyzer with a capacity of 270 kg and an accuracy of 100 g (Tanita SC-330S, Amsterdam, Netherlands). Body mass index (BMI) was calculated as weight (kg) divided by height squared (m^2^).

### Trunk endurance test

**Fig. 1:**
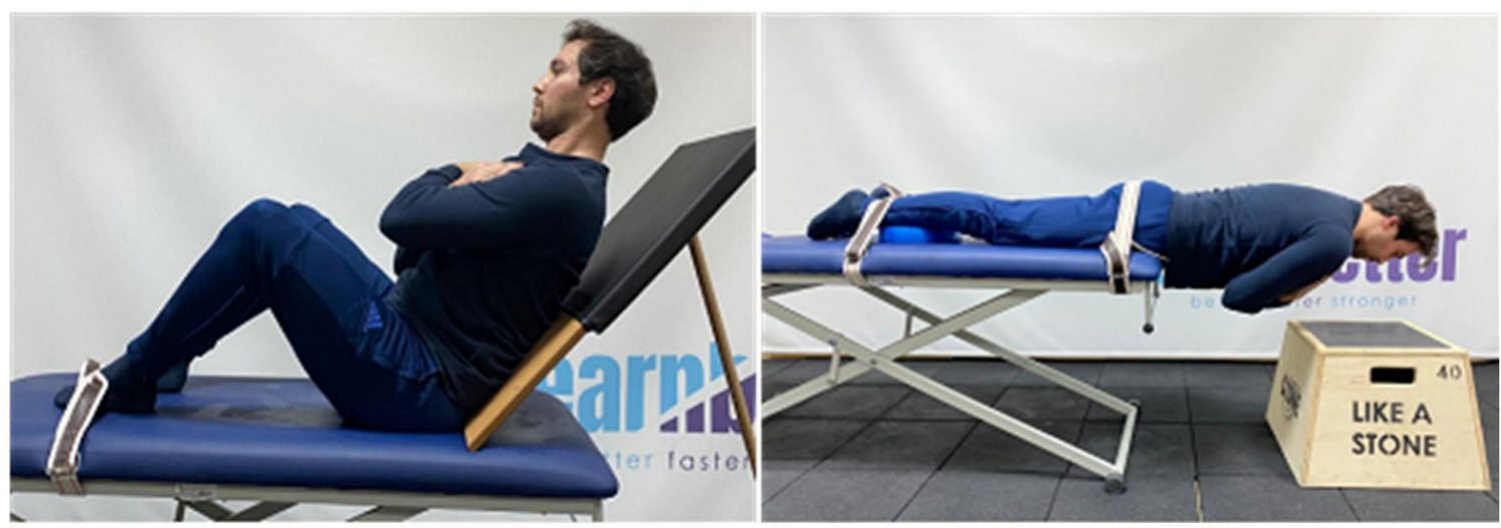
Trunk endurance test positions (left side: flexor test; right side: extensor test)

When assessing the rectus abdominis (RA) and obliques, a trunk flexion angle of 45° was preferred, which has a lower variance and higher reliability compared to 60°. For this angle, it is stated that the fatigue times of the target muscles can be analyzed more accurately by a mechanical extension of the power arm (Chen et al. 2003). The participant sat on the soft test bench and leaned against a support with the upper body at a 45° angle to the bench. The soles of the feet touched the bench and the hips and knees were bent at a 90° angle. A belt was tied around the participant’s feet to support them during the test. The participant’s hands were positioned crossed over the shoulders. The participant was instructed to maintain this position for as long as possible during the test. The duration of the test began when the upper body support was withdrawn 10 cm from the participant. The participant’s upper body angle was checked with a goniometer during the test and the test was terminated if the angle fell below 45° (Fig. 1, *left side*).

The Biering-Sorensen trunk extensor endurance test was used to measure the endurance performance of the participants’ back extensor muscles (Biering-Sørensen 1984). The participant was positioned in the prone position so that the anterior upper side of the iliac spine was in contact with the upper edge of the soft test stand. The pelvis, knees and ankles were secured with a belt to fix the participant to the bench. To avoid fatigue during positioning, a box was placed in front of the bench and about 25 cm below the bench for the participant to rest on. The test began with the participants placing their hands on opposite shoulders and being instructed to maintain the flat, horizontal position as much as possible throughout the test. The participant’s trunk angle was checked with a goniometer during the test and the test was terminated if the angle deviated 10° from the horizontal line (Fig. 1, *right side*).

### Surface electromyography measurements

Neuromuscular activation of the trunk muscles was evaluated using sEMG, with both anterior and posterior muscle groups analyzed. Specifically, the RA, external oblique (EO), and internal oblique (IO) were assessed in the anterior group, while the thoracic erector spinae (TES), lumbar erector spinae (LES), and multifidus (MULT) were evaluated in the posterior group. Raw EMG signals were recorded at a sampling rate of 1500 Hz using an 8-channel wireless telemetry system (Noraxon Desktop DTS, Noraxon USA, Scottsdale, Arizona) and analyzed with MyoMuscle MR 3.10 clinical application software (Noraxon USA). For measurements, wireless bipolar Ag/AgCl surface electrodes (diameter: 1 cm; inter-electrode distance: 2 cm) were used. Prior to electrode placement, the skin was prepared by shaving, cleaning with isopropyl alcohol wipes, and rubbing with a dry napkin to reduce skin impedance to below 10 kΩ. Electrodes were placed parallel to the muscle fibers to optimize signal quality.

### Anatomic positions of electrodes

**Fig. 2:**
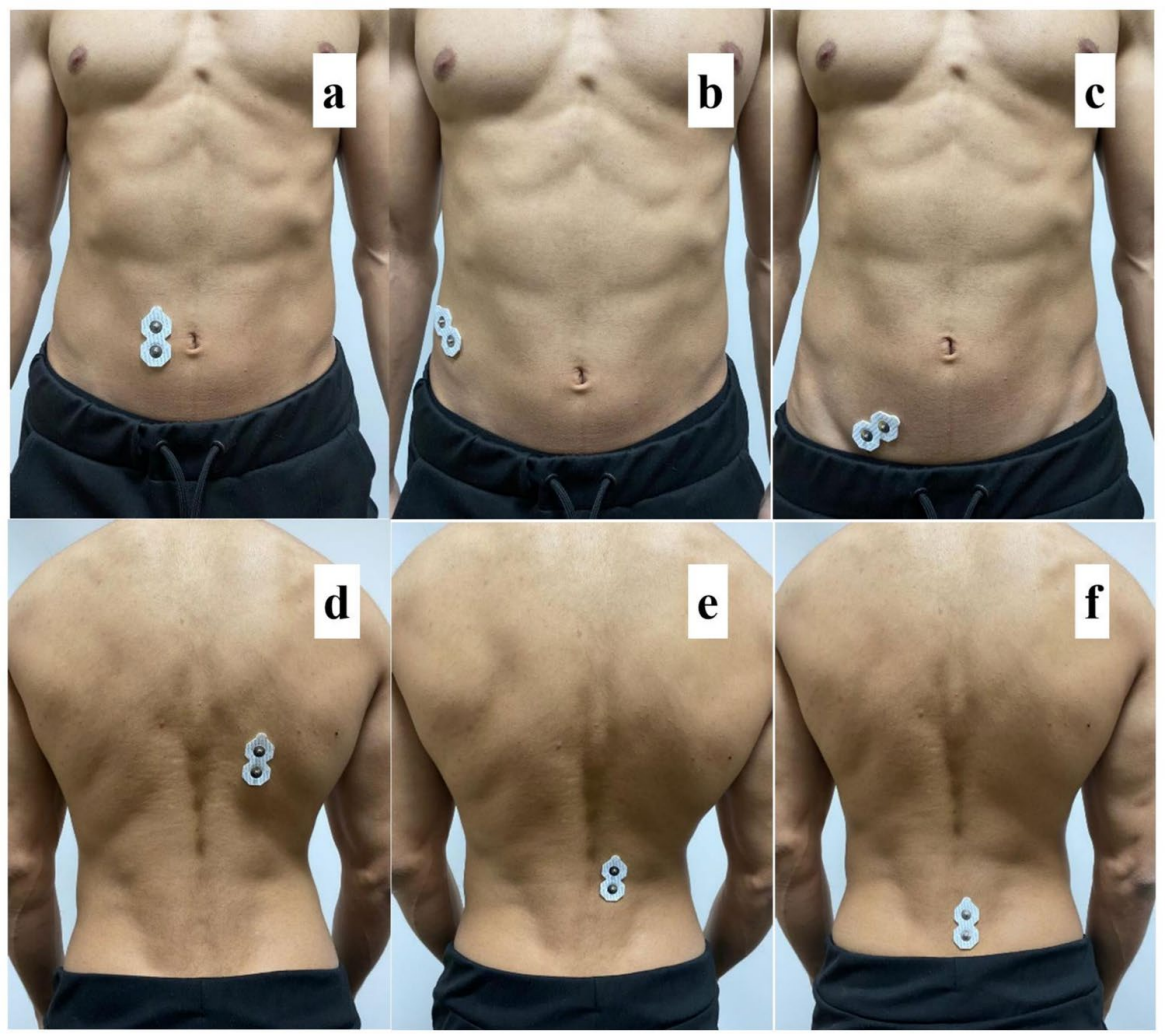
Location of electrodes (**a**: rectus abdominis; **b**: external oblique; **c**: internal oblique; **d**: torasic erector spinae; **e**: lumbar erector spinae; **f**: multifidus)

The electrodes were placed on the right side of the participants according to the SENIAM (Surface Electromyography for the Non-Invasive Assessment of Muscle) standards and recommendations (SENIAM, 2011). Rectus abdominis: It was placed 2 cm lateral to the umbilicus. External oblique: It was placed 1 cm below and 14 cm lateral to the umbilicus at a 45° angle to the area between the lowest level of the rib and the pubic bone. Internal oblique: The spina iliaca was placed 2 cm inferomedial to the most prominent point of the anterior superior. Thoracic erector spina: It was placed 5 cm lateral to the spinal protrusion at the level of T9. Lumbar erector spina: It was placed 3 cm lateral to the spinal protrusion at the level of L3. Multifidus: It was placed 2 cm laterally at the level between the spinal ridges L4-L5 (Fig. 2).

### Maximal voluntary contraction measurements

**Fig. 3:**
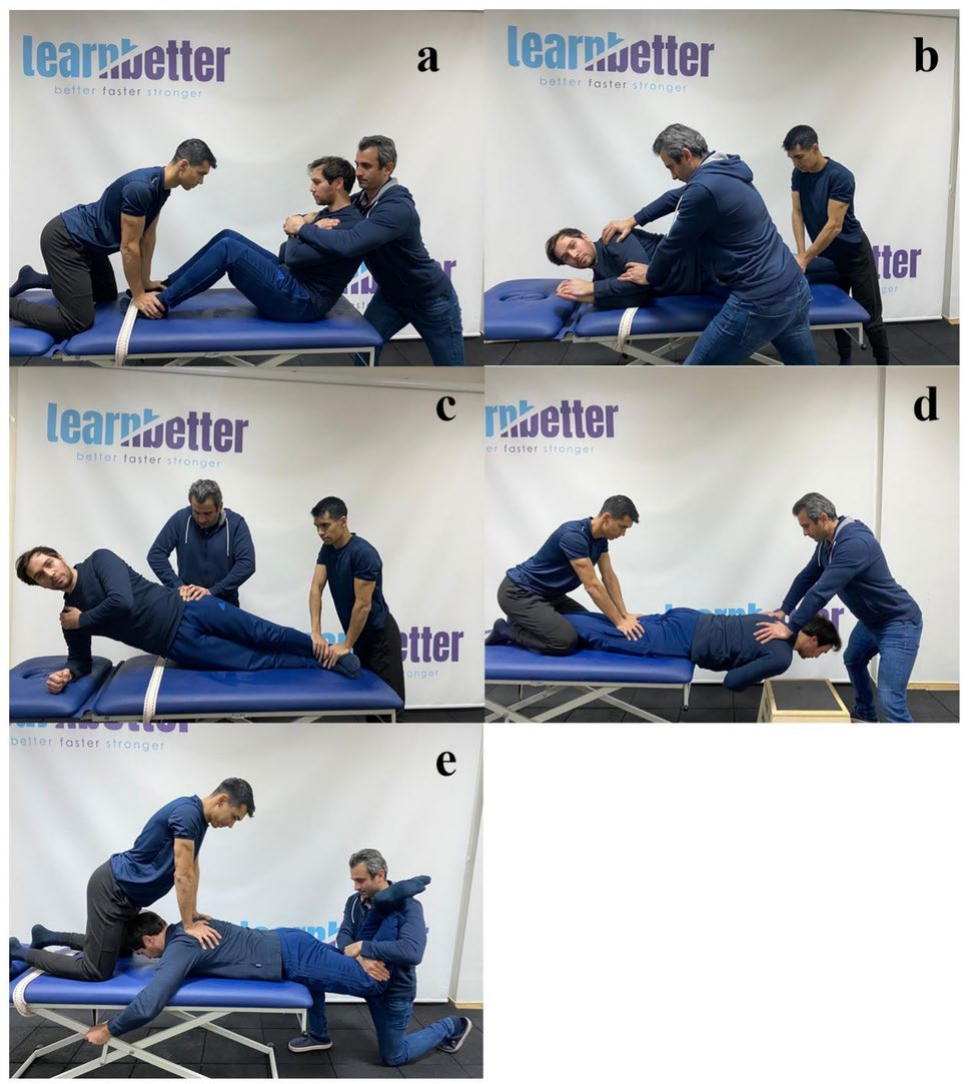
Maximal voluntary contraction measurements (**a**: Rectus abdominis; **b**: External obligue; **c**: Internal obligue; **d**: Torasik erector spinae; **e**: Lomber erector spinae and multifidus)

To normalize the raw EMG signals obtained during the endurance tests of the trunk flexors and extensors, measurements of maximal voluntary contraction (MVC) were performed before the endurance tests (Fig. 3). The measurements were performed with 3 repetitions of maximal isometric contractions of 5 s duration in different positions for the target muscle group(s). During the MVC tests, resistance was applied manually by the researchers. Rectus Abdominis: While the participant’s hips and knees were in a 90° flexion and the feet were bound in the arches, resistance was applied to the shoulders in the direction of extension and the participant was asked to maintain trunk flexion. External oblique: While the participant was lying on their side with knees bent and feet fixed, manual resistance was applied to the chest and arms while straightening along the frontal axis. Internal oblique: While the participant was in the lateral bridge position, maximum downward resistance was applied to the pelvis and the participant was asked to maintain the lateral bridge position. Thoracic erector spinae: After the participant assumed the position at the edge of the couch in prone position and at the level of the anterior superior iliac spine (SIAS), resistance was applied to the shoulders toward trunk flexion and the participant was asked to maintain the Sorensen position. Lumbar erector spinae and multifidus: From the level of the SIAS, the participant’s body was restrained with a strap and downward resistance was applied from the back of the knee while the participant lay prone on the couch with the knees flexed 90°. The participant was asked to maintain the starting position.

### Signal processing

The raw EMG signals obtained between the first 5 and 45 s of the trunk flexor and extensor endurance tests were analyzed after visual inspection and elimination of false signals. In the so-called normalized signal value analysis, the raw signal was first passed through a 20–500 Hz high-pass Butterworth filter and then through a 100-ms time window for motion artifact suppression or smoothing and the square root of the mean of the frames (filtered by the RMS method). The filtered signals were divided by the highest MVC values and interpreted as a percentage (Mok et al. 2015).

### Exercise procedure

The study was completed over eight weeks, consisting of a two-week familiarization phase followed by a six-week exercise intervention. The familiarization phase aimed to ensure that participants properly learned the key movement patterns required to achieve the desired neuromuscular adaptations. During this phase, participants performed the established movement patterns at 50% of the exercise volume to reduce injury risk and optimize motor learning (Da Silva-Grigoletto et al. 2019).

The subsequent 6-week intervention phase consisted of 12 exercise sessions (two sessions per week). Each session lasted approximately 30–34 min and followed a standardized format. The exercise programs were divided into four blocks, with movements designed to systematically target the trunk muscles across the sagittal, frontal, and transverse planes. This sequence was chosen based on evidence suggesting that spinal load and muscle activation increase significantly, especially during movements in these planes (Fountaine 2018).

To ensure consistent time under tension between groups, anti-movement exercises were performed for 30 s in an isometric hold, while dynamic exercises were performed for 12 repetitions within 30 s, paced with a metronome (45 bpm). At the end of each session, the OMNI-RES scale was used to evaluate perceived exertion, allowing for progressive overload monitoring. This scale has demonstrated a reliable intraclass correlation coefficient (ICC = 0.72–0.76), particularly in resistance band exercises (Colado et al. 2012).

### Anti-movement group

Resistance bands were used to achieve standardization (Sanctband, Sanctuary Health, Perak, Malaysia). In the first three weeks (sessions 1–6), participants used a light resistance band (pink – 70.60 Newtons at 100% stretch). In the second 3 weeks (sessions 7–12), they progressed to a medium resistance band (amber – 101.98 Newtons at 100% stretch), and a suspension device (TRX; Fitness Anywhere, Inc., San Francisco, USA) was introduced to increase the difficulty of plank-based positions. The resistance progression and exercise sequence were designed to promote trunk stability by challenging participants to resist external forces without compromising spinal alignment. Detailed information on the duration, intensity, and movement patterns of the program is provided in Table 1.

**Table 1:** Detailed program of the anti-movement exercise group

1. phase	2. phase		3. phase		4. phase
	1–6. Session	7–12. Session	1–6. Session	7–12. Session	
Warming -light jog -warming up the joints -dynamic streching	Anti-extension *Anterior Pallof Hold* *Pink Band* (2 × 30 s)	Anti- Extension *Anterior Pallof Hold* *Amber Band* (2 × 30 s)	Anti- Extension *Prone Plank* (2 × 30 s)	Anti- Extension *Suspended* *Prone Plank* (2 × 30 s)	Cooling -static streching
	Anti-flexion *Posterior Pallof Hold* *Pink Band* (2 × 30 s)	Anti-flexion *Posterior Pallof Hold* *Amber Band* (2 × 30 s)	Anti-flexion *Supine Plank* (2 × 30 s)	Anti-flexion *Suspended* *Supine Plank* (2 × 30 s)	
	Anti-lateral flexion *Side Plank Band Row* *Pink band* (2 × 30 s)	Anti-lateral flexion *Side Plank Band Row* *Amber Band* (2 × 30 s)	Anti-lateral flexion *Lateral Plank* (2 × 30 s)	Anti-lateral flexion *Suspended* *Lateral Plank* (2 × 30 s)	
	Anti-rotation *Supine Lying Pallof Hold* *Pink Band* (2 × 30 s)	Anti-rotation *Supine Lying Pallof Hold* *Amber Band* (2 × 30 s)	Anti-rotation *Pallof Hold* *Pink Band* (2 × 30 s)	Anti-rotation *Pallof Hold* *Amber Band* (2 × 30 s)	
Total time 4–5 min	Total time 11–12 min Inter-exercise 1 min. intensity(work:rest) 1/1 OMNI-RES: 6–7	Total time 11–12 min Inter-exercise 1 min. intensity(work:rest) 1/1 OMNI-RES: 7–8	Total time 11–12 min Inter-exercise 1 min. intensity(work:rest) 1/1 OMNI-RES: 6–7	Total time 11–12 min Inter-exercise 1 min. intensity(work:rest) 1/1 OMNI-RES: 7–8	Total Time 4–5 min

### Traditional dynamic group

During the first three weeks (sessions 1–6), participants completed level 1 exercises with lighter intensity. In the second 3 weeks (sessions 7–12), they progressed to level 2 exercises, which involved more challenging movement patterns and the addition of stability balls (TheraBand, The Hygenic Corporation, Akron, Ohio, USA). The progression was designed to improve trunk strength through controlled flexion, extension, and rotation movements. Detailed information on the TDG’s exercise structure is provided in Table 2.

**Table 2:** Detailed program of the traditional dynamic group

1. phase	2. phase		3. phase		4. phase
	1–6. Session	7–12. Session	1–6. Session	7–12. Session	
Warming -light jog -warming up the joints -dynamic streching	Anterior *Sit up* (2 × 12rep)	Anterior *Leg Raises* (2 × 12rep)	Anterior *Jack Knife* *Level 1* (2 × 12rep)	Anterior *Jack knife* *Level 2* (2 × 12rep)	Cooling -static streching
	Posterior *Straight Bridge* (2 × 12rep)	Posterior *Swiss Ball Bridge* (2 × 12)	Posterior *Back Extension* (2 × 12)	Posterior *Swiss Ball Reverse Extension* (2 × 12)	
	Lateral Flexion *Side Plank Hip Dips* (2 × 12rep)	Lateral Flexion *Swiss Ball Side Crunch* (2 × 12rep)	Lateral Flexion *Obligue V Up* *Level 1* (2 × 12rep)	Lateral Flexion *Obligue V Up* *Level 2* (2 × 12rep)	
	Rotational *Russian Twist* *Level 1* (2 × 12rep)	Rotational *Russian Twist* *Level 2* (2 × 12rep)	Rotational *Windshield Wiper* *Level 1* (2 × 12rep)	Rotational *Windshield Wiper* *Level 2* (2 × 12rep)	
Total Time 4–5 min	Total Time 11–12 min Inter-exercise 1 min. intensity(work:rest) 1/1 OMNI-RES: 6–7	Total Time 11–12 min Inter-exercise 1 min intensity(work:rest) 1/1 OMNI-RES: 7–8	Total Time 11–12 min Inter-exercise 1 min. intensity(work:rest) 1/1 OMNI-RES: 6–7	Total Time 11–12 min Inter-exercise 1 min. intensity(work:rest) 1/1 OMNI-RES: 7–8	Total Time 4–5 min

### Statictical analysis

Data analysis was conducted using SPSS version 25 (Statistical Package for Social Sciences). To compare baseline characteristics between the three groups, a one-way ANOVA was conducted for age, height, weight, body mass ratio, and body fat ratio. The significance level for all comparative tests was set at *p* < 0.05. Due to the violation of normality assumptions, comparisons within groups for dependent pairs were performed using the Wilcoxon signed-rank test. Effect sizes for the Wilcoxon test were calculated using the formula *r* = z/√n. The interpretation of effect sizes followed established guidelines: *r* < 0.3 was considered a small effect, 0.3 ≤ *r* ≤ 0.5 a medium effect, and *r* > 0.5 a large effect (Fritz et al. 2012) Percentage changes in mean values over time were calculated using the formula: post-test – pre-test) / pre-test*100″. Comparisons between independent groups were conducted using the Kruskal–Wallis test. Statistical significance was assessed with Bonferroni correction. Post hoc analyses were performed using the Mann–Whitney *U* test, with a corrected significance threshold of *p* < 0.017. For non-parametric data, effect sizes were calculated using eta-squared (*η*^2^) with the formula: (*η*^2^ = H − K + 1/(n − k). Eta-squared values were interpreted as follows: small = 0.01–0.06, medium = 0.061–0.14, and large > 0.14 (Tomczak and Tomczak 2014). Data are presented as median and interquartile range (P25–P75). Since the assumptions for multivariate analysis (e.g., multivariate normality and homogeneity of variances) were not met, a two-way permutational multivariate analysis of variance (PERMANOVA) was performed using the PAST 4.03 software. This non-parametric method was used to determine differences between groups, within groups, and interaction effects. Statistical significance for interaction effects was verified using Bonferroni-corrected p values. The analysis confirmed that no significant interaction effects were observed within groups.

## Results

Demographic characteristics were assessed, and the analysis indicated no significant differences between groups at baseline for age (*p* = 0.648), height (*p* = 0.216), weight (*p* = 0.546), body mass index (*p* = 0.420), or body fat percentage (*p* = 0.051) (Table 3).

**Table 3:** Baseline characteristics and descriptive statistics for the participants*

Groups	Age (years)	Height (m)	Weight (kg)	Body mass index (kg/m^−2^)	Body fat ratio (%)
AMG (n = 12)	22.66 ± 1.37	180.75 ± 6.87	73.30 ± 11.21	22.39 ± 2.78	13.00 ± 3.88
TDG (n = 12)	22.58 ± 1.08	176.58 ± 4.66	77.55 ± 9.00	24.03 ± 4.14	16.66 ± 3.35
CG (n = 12)	22.25 ± 0.96	177.83 ± 5.79	74.76 ± 8.07	23.61 ± 2.12	14.12 ± 3.51
*p* value	0.648	0.216	0.546	0.420	0.051

*Data are presented in mean ± standard deviation

*p* = the significant value of One-way ANOVA, *AMG* anti-movement group, *TDG* traditional dynamic group, *CG* control group

The analysis of the pre- and posttests for the interaction effect and the Kruskal–Wallis test in the study are shown in Table 4 as median and interquartile range (P25–P75). The Kruskal–Wallis test showed no significant differences between the pre-test values for all neuromuscular activation values. The post-test measurements showed significant differences (AMG vs. CG, *p* = 0.004) in the EO muscle (*η*^2^ = 0.17, *X*^2^ = 7.543, *p* = 0.023) and in the IO muscle (*η*^2^ = 0.30, *X*^2^ = 11.748, *p* = 0.003) (AMG vs. CG, *p* = 0.001). In addition, there was a significant difference between AMG vs. CG (*p* = 0.001) and TDG vs. CG (*p* = 0.001) in the LES muscle (*η*^2^ = 0.37, *X*^2^ = 14.261, *p* = 0.001). Finally, there was a significant difference between AMG vs. CG (*p* = 0.010) and TDG vs. CG (*p* = 0.014) in the MULT muscle at post-test measurements (*η*^2^ = 0.19, *X*^2^ = 8.269, *p* = 0.016) (Table 4).

**Table 4:** Comparison of the nMVC values in relation to the trunk flexor and trunk extensor muscle endurance test between the groups by time*

Variables (MVC%)	Anti-movement group	Traditional dynamic group	Control group	Interection effect (*p*_1_)	Inter-group (*p*_2_)
	Median (P25–P75)	Median (P25–P75)	Median (P25–P75)		
RA_Pre_	23.35 (18.62–60.45)	24.45 (11.52–45.97)	29.2 (11.92–42.87)	F = 1.832 p = 0.170	*X*^2^ = 0.464 *p* = 0.793
RA_Post_	14.6 (10.57–21.60)	18.3 (8.12–33.57)	32 (13.50–41.70)		*X*^2^ = 3.993 *p* = 0.136
EO_Pre_	37.15 (32.47–42.32)	34.55 (24.12–50.15)	41.65 (30.02–53.40)	F = 1.221 p = 0.304	*X*^2^ = 0.978 *p* = 0.613
EO_Post_	25.75 (14.95–29.52)	25.05 (16.95–39.57)	42 (28.75–47.67)		*X*^2^ = 7.543 *p* = 0.023
IO_Pre_	35.05 (27.57–55.45)	30.5 (15.97–53.00)	42.6 (27.77–74.52)	F = 1.781 p = 0.175	*X*^2^ = 2.066 *p* = 0.356
IO_Post_	19.85 (13.40–26.37)	22.95 (11.75–37.02)	46.5 (30.17–65.75)		*X*^2^ = 11.748 *p* = 0.003
TES_Pre_	22.35 (17.62–25.60)	18.8 (13.55–23.85)	17.45 (14.12–22.80)	F = 0.911 p = 0.43	*X*^2^ = 2.051 *p* = 0.359
TES_Post_	15.9 (12.97–22.02)	15.45 (12.25–18.25)	18.8 (15.72–25.65)		*X*^2^ = 3.194 *p* = 0.202
LES_Pre_	31.8 (26.50–36.12)	28.35 (20.40–31.02)	30.75 (26.12–37.77)	F = 3.784 p = 0.029	*X*^2^ = 3.673 *p* = 0.159
LES_Post_	20.95 (19.05–26.25)	21.2 (16.47–27.05)	35.75 (27.52–37.22)		*X*^2^ = 14.261 *p* = 0.001
MULT_Pre_	34.1 (25.17–37.75)	29.35 (20.52–35.07)	30.6 (26.82–34.22)	F = 3.005 p = 0.057	*X*^2^ = 2.012 *p* = 0.366
MULT_Post_	24.25 (19.50–29.02)	20.65 (17.65–28.30)	33.6 (25.10–38.90)		*X*^2^ = 8.269 *p* = 0.016

*Data are presented in median and interquartile range (P25–P75)

*RA* rectus abdominis, *EO* external obligue, *IO* internal obligue, *TES* thoracic erector spinae, *LES* lumbar erector spinae, *MULT* multifidus, *p*_*1*_ the significant value of PERMANOVA interection effect, *p*_*2*_ the significant value of Kruskal–Wallis Test

PERMANOVA analysis showed that the change and interaction effect between groups as a function of time was statistically significant only for the LES muscle (*F* = 3.784, *p* = 0.029). There was a statistically significant difference between the AMG vs. CG groups (*p* = 0.016) and between the TDG vs. CG groups (*p* = 0.004). However, the differences in neuromuscular activation of the other muscles were not significant (*p* < 0.05) (Table 4).

**Fig. 4:**
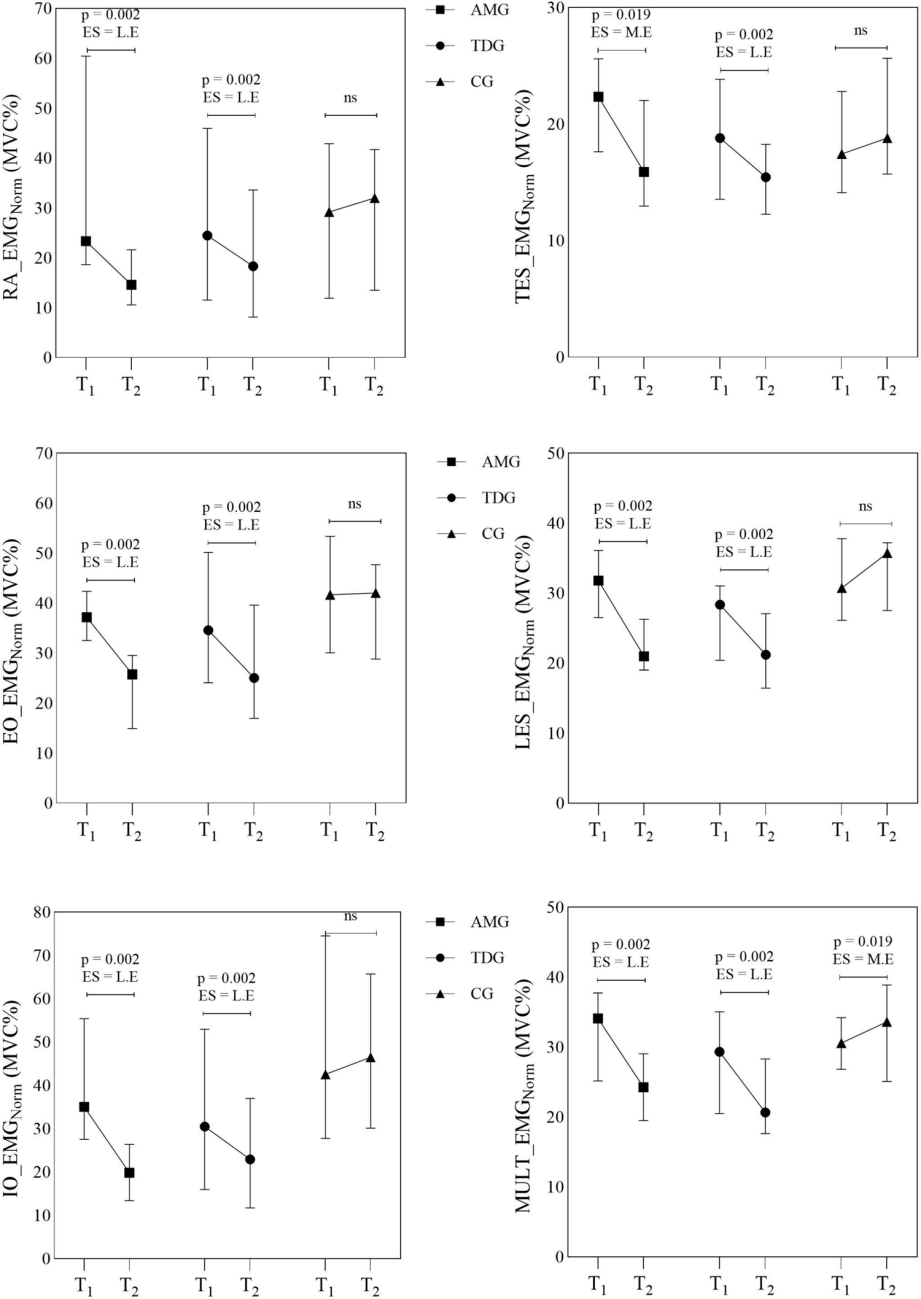
Results within the group (Wilcoxon signed ranks test) for anti-movement (AMG), traditional dynamic (TDG) and control group (CG). L.E: large effect; M.E: moderate effect; ns: not significant; EMGNorm: normalized activation

Figure 4 shows that there was a statistically significant difference between the pre-test and the post-test as a function of time for the two training groups (*p* < 0.05; effect size *r* range = 0.48 − 0.63). Although an increase in MULT muscle was noted in the control group, it was likely a negative increase (7%, *p* = 0.019; *r* = 0.48).

**Fig. 5:**
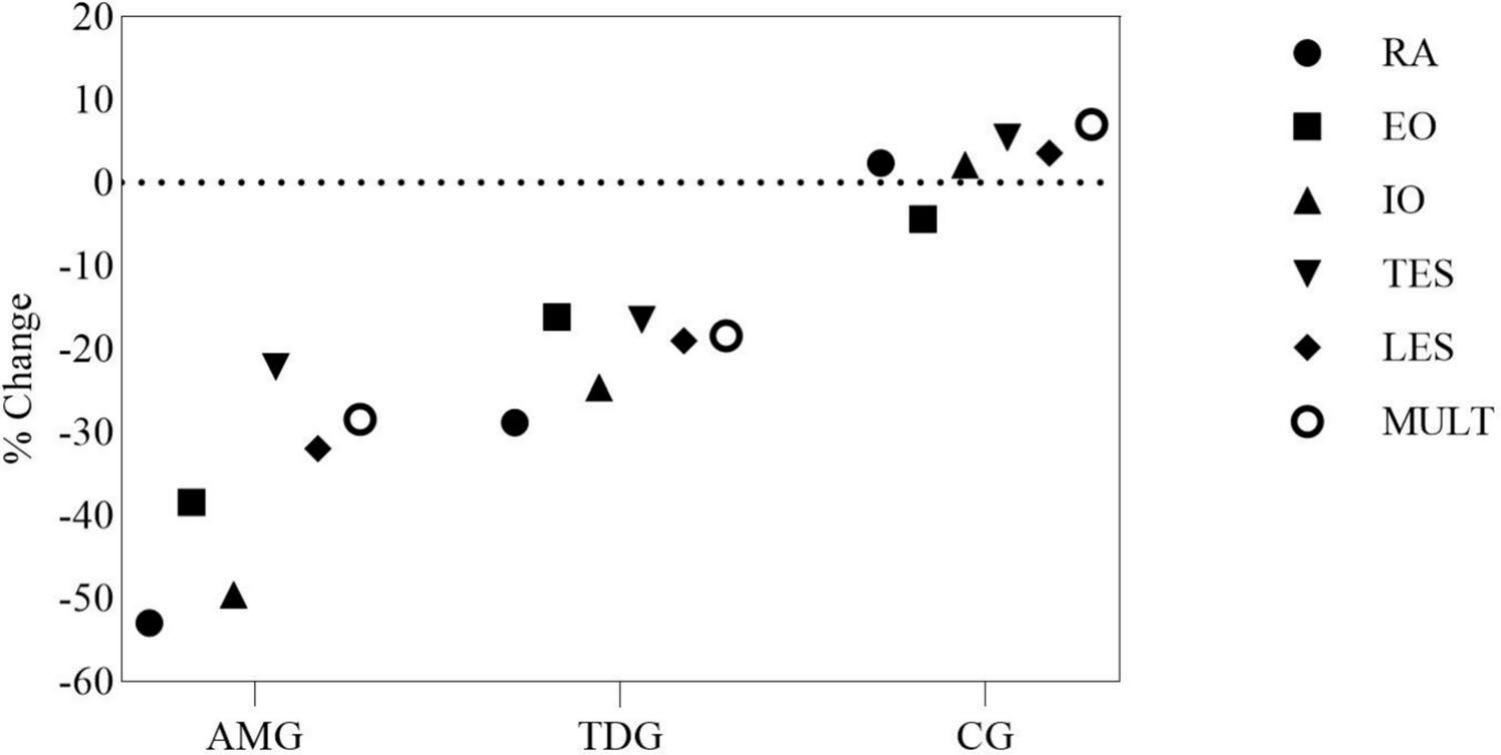
Percentage changes pre- to post- training in normalized neuromuscular activation for anti-movement (AMG), traditional dynamic (TDG) and control group (CG)

The percentage change for AMG, TDG and CG in neuromuscular activation is shown in Fig. 5. The range of neuromuscular changes after AMG training was a clear improvement in the RA (AMG: 53% vs. TDG: 28.82%), EO (AMG: 38.44% vs. TDG: 16.18%), IO (AMG: 49.45% vs. TDG: 24.65%), TES (AMG: 22.17% vs. TG: 16.50%), LES (AMG: 31.97% vs. TDG: 19.04%) and MULT (AMG: 28.45% vs. TDG: 18.45%).

## Discussion

To our knowledge this was the first study to investigate the effects of anti-movement vs. traditional dynamic core-training program on neuromuscular activation. Compared to the CG, there was only a significant difference in the oblique muscles in the AMG group. However, a neuromuscular improvement was observed in the TES and the multifidus muscles in both training groups. The change and interaction effect between groups as a function of time was statistically significant only for the LES muscle for both training groups.

Training practices have significant effects on the muscle activation patterns. The rational reasons for these effects are the changes in muscle conduction velocity, motor unit involvement, motor unit synchronization, and the level of fibril activation (Casolo et al. 2020; Tumkur Anil Kumar et al. 2021). The improvements in EMG reductions observed in our study are consistent with the existing literature. One study found that after 8 weeks of endurance training for the wrist flexor muscles, there was a decrease in EMG amplitude values, possibly due to the development of motor learning and strength performance (Oliveira and Gonçalves 2009). In another study, it was found that rowing training (10 sessions) saves energy by reducing the mechanical work required, which leads to a reduction in the activation level of the vastus lateralis and biceps brachii muscles (Lay et al. 2002).

There are some rational explanations for the decrease in activation that occurs after exercise with the same workload. It has been suggested that the tendency to reduce activation levels is related to the synchronization of motor units that enables motor learning (Madeleine et al. 2003). Seidel et al. (2017) found that motor learning reduced EMG activity of the tibialis anterior muscle by approximately 30% during static balance exercises. Metabolic efficiency can be achieved with the motor-learning strategy and a reduction in activation levels can be observed (Huang et al. 2012). In our study, both training groups may have made motor-learning gains in the core muscles they used during their training program.

Another possible explanation for the decrease in signal amplitude values is the mechanism of involvement of the motor unit required by the muscle. Studies generally show an increase in EMG signal amplitudes as a function of action potential during progressively increasing submaximal exercise (Mathur et al. 2005; Cao et al. 2017). It is said that the increase in amplitude is an interpretable parameter related to the rate of motor unit involvement (Li et al. 2022). In addition, De Luca explained that the increase in RMS value due to fatigue is due to the compression of the EMG signal to lower frequencies in the spectrum due to reduced conduction velocity (De Luca 2008). With the increase in external force, compensation by the muscles takes place to sustain the movement and an increase in signal values is observed. In our study, results were obtained in the direction of a decrease in the need to participate in the activity of motor units with greater energy generating capacity after training performs. In this context, it is also found that EMG signal amplitude can increase with fatigue and this increase is related to the high-threshold fibril involvement required by the pool of motor units to maintain force production (Sundstrup et al. 2012; Asmussen et al. 2018). A study showed that the RMS values of trained hikers increase less during an isometric fatigue test than those of untrained hikers. This result was explained by the ability of trained subjects to delay the activation of new motor units (Boyas et al. 2009).

It is assumed that the decrease in the activation values of the six core muscles in the anti-training group in our study can be explained by the size principle already explained in the literature. According to this principle, it is assumed that when the muscle is initially activated, units of small size and low degree of tension are fired first, and that as the force of muscle contraction increases, progressively larger units are involved (Hodson-Tole and Wakeling 2009). The fact that the quadriceps and hamstring muscles generate higher EMG activity during low and high resistance training can also be explained by the size principle (Schoenfeld et al. 2014). In our study, the change in neuromuscular activation scores was extremely dominant with AMG (~ 22%–53% decrease) compared to TDG (~ 16%–29% decrease). The percentage changes of the two training groups, especially in the deep muscles (IO, AMG ~ 50% vs. TDG ~ 25%; Multifidus, AMG ~ 28% vs. TDG ~ 18%), show the effectiveness of anti-movement training on the deep core muscles. Therefore, it can be concluded that the AMG group has a more beneficial effect in terms of mechanical efficiency and can reduce the need for larger fibrillar muscles by delaying fatigue.

The endurance capacity of the trunk has a protective effect on the passive structures of the spine and that the stabilization requirements of the spine are more related to endurance and control elements than to strength (Evans et al. 2007). It has been observed that before any movement of the limbs, the local muscles associated with the vertebrae are active before the main transport or agonist muscles (Fredericson and Moore 2005). It can therefore be said that adequate local stabilization is particularly important for athletic performance and injury prevention. Local muscles are involved in activity before the global muscles and affect minimal movements. For this reason, the first step in planning an anti-movement workout can be to activate the local system as a strategy to stabilize the lumbo-pelvic region.

## Conclusion

In conclusion, the significant results obtained in this study in terms of amplitude values support previous studies and confirm the predicted activation changes. In terms of muscle activation, both exercise approaches are applicable, but anti-movement exercises are recommended because of their effects, such as possible spinal health and deep muscle excitation. Research suggests that anti-movement exercises can optimize neuromuscular activation of trunk muscles so that movement can be maintained with less effort. Because it is extremely important to keep the vertebrae in a neutral zone during trunk muscle exercises, this may be an alternative to exercises based on bending and twisting. Anti-movement exercises can be a user-friendly training method as they do not contribute to the development of lower back pain and hernias. Future studies can investigate the effect of anti-movement exercises on activities of daily living in patients who need therapeutic exercises, such as patients with non-specific low-back pain and patients with post-operative hernias in the rehabilitation process.

## Data Availability

The data that support the findings of this study are available from the corresponding author upon reasonable request.

## Abbreviations

EMG: Electromyography
MVC: Maximal voluntary contraction
AMG: Anti-movement group
TDG: Traditional dynamic group
RA: Rectus abdominis
EO: External obligue
IO: İnternal obligue
TES: Thoracic erector spinae
LES: Lumbar erector spinae
MULT: Multifidus
